# P02-005 - Overlap of FMF and HIDS in one Arabic family

**DOI:** 10.1186/1546-0096-11-S1-A112

**Published:** 2013-11-08

**Authors:** T Moussa, B Aladbe, A Aly, R Taha, H El-Shanti, B Fathalla

**Affiliations:** 1 Pediatrics, Hamad General Hospital, Doha, Qatar; 2 Pediatrics/Rheumatology, Hamad General Hospital, Doha, Qatar; 3 Medical Genetics, Shafallah Medical Genetics Center, Doha, Qatar

## Introduction

Familial Mediterranean Fever (FMF) is commonly reported in Arabs, whereas Hyper-IgD syndrome (HIDS) is rare. Moreover, the simultaneous presence of *MEFV* and *MVK* mutations segregating in the same family is exceedingly rare. We report here an Arabic family in whom a combination of complex *MEFV* mutations and an *MVK* mutation segregate producing variable clinical phenotypes.

## Case report

An 8-year-old female presented with episodes of fever, abdominal pain, vomiting, and arthralgia lasting 3-5 days for 1-year duration suggestive of FMF. Atypical FMF features included longer episodes of fever and partial response to colchicine. Family history revealed HIDS in an 18 years old brother. He presented with episodes of fever, abdominal pain, vomiting, diarrhea, skin rash, lymphadenopathy, and febrile seizures since 1 year of age and was treated as clinical FMF with colchicine for 4 years with poor response. Genetic testing for HIDS done at 7 years of age showed homozygosity of V377I mutation. He was not responsive to Statins but became asymptomatic after puberty. At 17 years of age he developed short episodes of fever and abdominal pain more consistent with FMF. He was responsive to bursts of prednisone during episodes but not compliant with colchicine due to severe diarrhea.

Genetic testing was done for both patients and asymptomatic family members by sequence analysis of entire *MEFV* and *MVK* as well as *TNFRSF1A*, *PSTPIP1*, *IL1RN* and *LPIN2* coding regions and splice sites. Asymptomatic parents are carriers of V377I/- *MVK* mutation. The father is a compound heterozygote for two complex *MEFV* mutations, E148Q/P369S/R408Q and E167D/F479L whereas the mother is a compound heterozygote for M680I and the complex allele E148Q/P369S/R408Q. Both of our patients are homozygous for V377I *MVK* mutation, the girl is compound heterozygote for E148Q/P369S/R408Q and E167D/F479L *MEFV* mutations whereas the boy is compound heterozygote for E148Q/P369S/R408Q and M680I.

## Discussion

The presence of concomitant mutations in different genes of monogenic autoinflammatory diseases (AID) could act as potential disease modifiers. Clinical implications to such combinations are not clear but may explain overlap or atypical clinical features. Such combinations have been scarcely reported including *TNFRS1A* and *MEFV* mutations, *MVK* and *TNFRSFA1* mutations, and *CIAS1* and *MEFV* mutations. Utilizing the diagnostic score and proposed diagnostic algorithm for molecular analysis of hereditary AID with periodic fever in children could have possibly resulted in genetic testing for one AID and missed such combinations. Our reported family does suggest that multiple mutations/variants in AID genes can occur in the same patient and could potentially influence the clinical presentation and response to treatment.

## Disclosure of interest

None declared.
